# Choroid plexus genes for CSF production and brain homeostasis are altered in Alzheimer’s disease

**DOI:** 10.1186/s12987-018-0120-7

**Published:** 2018-12-12

**Authors:** Shawn Kant, Edward G. Stopa, Conrad E. Johanson, Andrew Baird, Gerald D. Silverberg

**Affiliations:** 1 Department of Pathology (Neuropathology Division), Warren Alpert Medical School at Brown University, Providence, RI 02903 USA; 2 Department of Neurosurgery, Warren Alpert Medical School at Brown University, Providence, RI 02903 USA; 3 Department of Surgery, University of California San Diego, La Jolla, CA USA; 4 Department of Neurosurgery, Stanford University, 710 Frenchmans Rd, Stanford, CA 94305 USA

**Keywords:** CSF production, Homeostasis, Blood–CSF barrier, Blood–brain barrier, Choroid plexus Gene Expression Omnibus, Solute carrier families, Choroidal tight junctions, Barrier permeability, Choroid plexus transcriptome, Beta amyloid, Mitochondria

## Abstract

**Background:**

The roles of the choroid plexus (CP) and cerebrospinal fluid (CSF) production have drawn increasing attention in Alzheimer’s disease (AD) research. Specifically, studies document markedly decreased CSF production and turnover in moderate-to-severe AD. Moreover, reduced CP function and CSF turnover lead to impaired clearance of toxic metabolites, likely promote neuroinflammation, and may facilitate neuronal death during AD progression. We analyzed CP gene expression in AD compared with control subjects, specifically considering those genes involved with CSF production and CP structural integrity.

**Methods:**

The Brown-Merck Gene Expression Omnibus (GEO) database (CP transcripts) was mined to examine changes in gene expression in AD compared to controls with a focus on assorted genes thought to play a role in CSF production. Specifically, genes coding for ion transporters in CP epithelium (CPE) and associated enzymes like Na–K-ATPase and carbonic anhydrase, aquaporins, mitochondrial transporters/enzymes, blood–cerebrospinal fluid barrier (BCSFB) stability proteins, and pro-inflammatory mediators were selected for investigation. Data were analyzed using t test p-value and fold-change analysis conducted by the GEO2R feature of the GEO database.

**Results:**

Significant expression changes for several genes were observed in AD CP. These included disruptions to ion transporters (e.g., the solute carrier gene SLC4A5, p = 0.004) and associated enzyme expressions (e.g., carbonic anhydrase CA4, p = 0.0001), along with decreased expression of genes involved in BCSFB integrity (e.g., claudin CLDN5, p = 0.039) and mitochondrial ATP synthesis (e.g., adenosine triphosphate ATP5L, p = 0.0004). Together all changes point to disrupted solute transport at the blood–CSF interface in AD. Increased expression of pro-inflammatory (e.g., interleukin IL1RL1, p = 0.00001) and potential neurodegenerative genes (e.g., amyloid precursor APBA3, p = 0.002) also implicate disturbed CP function.

**Conclusions:**

Because the altered expression of numerous transcripts in AD-CP help explain decreased CSF production in AD, these findings represent a first step towards identifying novel therapeutic targets in AD.

**Electronic supplementary material:**

The online version of this article (10.1186/s12987-018-0120-7) contains supplementary material, which is available to authorized users.

## Background

Alzheimer’s disease (AD) is a neurodegenerative disorder marked by cognitive, memory and behavioral impairment that significantly interferes with social and occupational functioning. It is an incurable disease, at present, with a long preclinical period and progressive course. In AD, amyloid-beta (Aβ) peptide plaques develop in the hippocampus, and in other areas of the cerebral cortex. Whether plaques themselves cause AD or whether they are a by-product of the AD process remains unknown.

The roles of the blood–cerebrospinal fluid (CSF) barrier (BCSFB) and CSF itself in the pathogenesis of AD are receiving increasing attention [1–5]. Like the blood–brain barrier (BBB), the BCSFB functions as a transport interface, facilitating exchange of solutes and H_2_O between plasma and CSF [6]. The four choroid plexuses (CP), one in each ventricle, are the anatomic substrate of the BCSFB and are responsible for more than 60–75% of CSF production [7] with the remainder produced by the ventricular ependyma and BBB. At the cellular level, CP epithelium (CPE) cells are continuous with the ventricular ependymal layer and have apical microvilli. However, unlike the tight junctions of the BBB between capillary endothelial cells, the tight junctions of the BCSFB are located only at the apical portion of the CPE. In humans, normal CSF secretion ranges from 400 to 600 ml/day [1, 7].

Production of CSF by CP occurs in two major sequential steps: passive fluid ultrafiltration across CP capillaries and active ion transport across the CPE [7, 8]. A pressure gradient first filters plasma from choroidal capillaries into the interstitial compartment of the CP [9]. Carbonic anhydrases catalyze conversion of H_2_O and CO_2_ to H^+^ and HCO_3_^−^ ions. Ion co-transporters and exchangers translocate Na^+^, K^+^, Cl^−^, and HCO_3_^−^ ions from the interstitial fluid across the basolateral membrane into the CPE cell and then, after cytoplasmic swirling, across the apical membrane into the ventricles by energy-dependent active transport [10–15]. Water flows across the CPE from plasma to ventricular lumen (e.g., through aquaporins) in concordance with prevailing osmotic solute gradients [16].

Considerable neurodegeneration research has focused on increased BBB permeability and decreased efficiency of Aβ peptide clearance across the BBB in aging and in AD [17–19]. Interestingly, just as in the BBB, tight junctions in CP lose integrity during AD progression. This is consistent with increased paracellular permeability and BCSFB breaching [20, 21]. Indeed, many of the structural changes that occur in the AD CP are among the first signs of the disease in a subset of patients, including extensive atrophy of the CPE that resembles analogous changes seen in accelerated aging [20].

Clinical evidence also points to significantly decreased CSF production rates in moderate-to-severe stages of AD versus normal controls [1]. For example, CSF production is ~ 1/2 normal and CSF turnover, defined as the volume of CSF produced in 24 h divided by the volume of the CSF space, is reduced by threefold, from four times to once per day in AD subjects compared to age-matched controls [1, 2].

Current theory suggests that CSF hydrostatic pressure initially rises during early AD before falling again in later-stage AD, as reduced CSF production outpaces the decreased CSF absorption [22]. Decreased CSF production and turnover in AD have significant consequences for removing toxic metabolites from the CNS. For example, altered CPE cholesterol metabolism may affect Aβ clearance from CSF [23]. Diminished CSF production also decreases the ability of CP-secreted transthyretin (TTR) to circulate throughout the CNS via the CSF and bind to and stabilize Aβ deposits [20].

Taken together, these findings highlight the need to elucidate gene-linked predisposition to pathology in CP, CSF, and BCSFB, and how modified transcript production adversely affects CP–CSF solute homeostasis and neuropathology. Of particular interest are the specific genetic factors behind impaired CSF production and turnover in AD. We investigated genes known to be involved in these processes for expression differences between healthy and AD-affected CP. These data inform on genes that most strongly affect the outcome of CSF production and potentially have the strongest impact on AD pathology progression in the CP–CSF–brain system.

## Methods

A choroid plexus Gene Expression Omnibus (GEO) database archived at https://www.ncbi.nlm.nih.gov/geo/query/acc.cgi?acc=GSE110226 under the GEO accession number GSE110226 was mined for gene expression differences between choroid plexus from control and AD subject brains.

### Subject matter

The Brown-Merck database was created using transcriptome-wide Affymetrix microarray assays (Affymetrix, Santa Clara, CA, USA) to examine gene expression via extracted RNA from human CP tissue samples [24]. RNA was extracted with TRIzol reagent by the Thermo-Fisher protocol (Thermo-Fisher, Grand Island, NY, USA). Lateral ventricle CP tissue samples were taken from six control cases, seven late-stage AD cases, four frontotemporal dementia cases, and three Huntington’s disease cases. Tissue samples were post-mortem, mean post mortem interval (PMI) of 22 h for controls, 17 h for AD. Until processing could occur, tissues were snap frozen in liquid nitrogen and stored at − 80 °C at the Brown University Brain Tissue for Neurodegenerative Disorders Resource Center. For this study, we only mined data for gene expression level differences between control and AD cases. AD cases were slightly older than controls. This is an advantage because older people and those with early AD often show no significant differences in gene expression (personal observation by author EGS from prior gene studies).

Genes of interest (GoI) were selected based on their known or purported significance to CP function and/or CSF production. For example, genes and mRNA that express solute transporters in CPE are integral to CSF production [25]. Specifically, genes involved in the active transport of ions (along with the obligatory transport of H_2_O) from blood to the ventricular lumen are the final pathway of CSF production; these are considered rate-limiting genes. Therefore we focus considerable attention on genes for Na–K-ATPase, the Na–K–Cl cotransporter, and other components of this final secretory pathway.

GoI involved in mitochondrial ATP synthesis and ion transport were also selected. Without sufficient mitochondrial energy production, the active ion transporters in CP cannot operate normally. Intracellular bicarbonate production facilitates HCO_3_^−^-dependent Na^+^ and Cl^−^ exchange across the CPE [26–29]. In this vein, we consider the carbonic anhydrase family, along with several members of the solute carrier (SLC) gene family responsible for HCO_3_^−^ exchange and transport. Genes encoding BCSFB structural elements for maintenance of regulated solute transport were considered as well, along with pro-inflammatory and neurodegenerative genes capable of damaging the BCSFB.

### Statistical methods

Gene expression differences between control and AD samples in the database were determined using the GEO2R search feature of the GEO database. This approach creates different groups of samples based on unifying characteristics for each group. GEO2R then generates statistics for gene expression comparisons between groups. Examining visual profiles depicting mRNA expression levels for each sample in the AD versus control groups generated by GEO2R, allowed visualization of upregulation versus downregulation of various genes in AD compared with control CP tissue.

The Brown-Merck database was first mined to identify which specific genes involved in CSF production and CP function differed in expression between control and AD samples. Separate tables were then composed for genes that were upregulated, downregulated or unchanged in AD. Significance was assessed with t-test p value analyses of each gene. An α of < 0.05 was deemed significant. The sign of the moderated t statistic of each gene confirmed the direction of gene expression changes (upregulation or downregulation) seen in the visual mRNA expression profiles.

Fold changes to quantify the magnitude of gene expression differences between control and AD groups were determined through GEO2R log base 2 fold change (log2 FC) analysis, reported as log2FC values. GEO2R took the difference between log2 of the gene expression value of a given gene in the control group and log2 of the gene expression value of that same given gene in the AD group to produce log2FC in the tables. Hence positive log2FC values signify downregulation in AD relative to control and negative log2FC values signify upregulation in AD relative to control. Additional file 1 gives actual gene expression values for each gene studied.

## Results

We investigated expression levels of several different genes and gene families that are purported to impact CP structural integrity and CSF production. The SLC family of genes, for example, appears responsible for considerable ion and H_2_O transport across CPE [25]. Within the SLC group, there was diversified expression in control versus AD. Certain genes for HCO_3_^−^ exchange and co-transport (SLC4 subfamily) had decreased expression in AD, including SLC4A10 (p = 0.028, log2FC = 0.039) and SLC4A5 (p = 0.004, log2FC = 1.12). Other SLC genes with decreased expression include the Na–K–Cl co-transporters SLC12A1 (p = 0.035, log2FC = 1.05) and SLC12A2 (p = 0.005, log2FC = 0.38). Table 1 compiles downregulated CP genes in the Brown-Merck database relevant to CSF production, energy production and CP structural integrity. Included in each table is the fold-change (log2 FC) in expression between AD and control.

**Table 1:** Genes downregulated in AD CP

Gene symbol	Gene name	p-value	t-value (control relative to AD)	Log2 fold change
SLC family				
SLC26A2	Solute carrier family 26 member 2; sulfate transporter	0.0000313	5.88360095	1.43830952
SLC5A5	Solute carrier family 5 member 5; sodium/iodine cotransporter	0.0000375	5.78520397	0.75760238
SLC4A10	Solute carrier family 4 member 10: Na-dependent Cl–HCO3 exchanger	0.0275	2.44489236	0.39339524
SLC4A5	Solute carrier family 4 member 5: Na–HCO3 exchanger	0.00366	3.44425553	1.1215619
SLC12A1	Solute carrier family 12 member 1: NKCC2 Na–K–Cl cotransporter	0.0353	2.31563648	1.05009524
SLC12A2	Solute carrier family 12 member 2: NKCC1 Na–K–Cl cotransporter	0.00517	3.27527462	0.38004286
Na–K ATPase family				
ATP1A2	Na–K ATPase transporting subunit alpha 2	0.0387	2.26741686	0.50910238
ATP1B1	Na–K ATPase transporting subunit beta 1	0.0429	2.21376353	0.29179286
Carbonic anhydrase family				
CA4	Carbonic anhydrase 4	0.000139	5.08319761	0.63055238
CA2	Carbonic anhydrase 2	0.0222	2.55323972	0.30442619
CA3	Carbonic anhydrase 3	0.0391	2.26309122	0.58876429
ATP synthase family				
ATP5L	ATP synthase, H+ transporting, mitochondrial Fo complex subunit G	0.000419	4.51769377	0.44282857
ATP23	ATP23 metallopeptidase and ATP synthase assembly factor homolog	0.000780	4.20573756	0.62625
ATP5G3	ATP synthase, H+ transporting, mitochondrial Fo complex subunit C3 (subunit 9)	0.00154	3.86838238	0.65084286
ATPAF1	ATP synthase mitochondrial F1 complex assembly factor 1	0.00269	3.59573018	0.56412619
ATP5B	ATP synthase, H+ transporting, mitochondrial F1 complex, beta polypeptide	0.0107	2.91564259	0.45252381
Tight junction claudins				
CLDN18	Claudin-18	0.0105	2.92789759	0.46640476
CLDN11	Claudin-11	0.0190	2.63115902	0.64749762
CLDN5	Claudin-5	0.0399	2.25235984	0.70879524
Others				
APP	Amyloid beta precursor protein	0.0144	2.77007349	0.41097857

Positive t values for comparisons represent gene changes decreased in AD relative to control (i.e. increased in control relative to AD). The same is true for positive log2FC values

Among the upregulated SLC genes in AD CP were mitochondrial Fe transporters: SLC25A37 (p = 0.0001, log2FC = − 0.64) and SLC25A28 (p = 0.0001, log2FC = − 0.64). Other ion-transporters, like the KCC4 K–Cl cotransporter SLC12A6 (p = 0.026, log2FC = − 0.67) and the organic anion clearance transporter SLCO4A1 (p = 0.0001, log2FC = − 1.85), were upregulated in AD. Table 2 lists upregulated genes, from the Brown-Merck database, involved in CSF production.

**Table 2:** Genes upregulated in AD  CP

Gene symbol	Gene name	p-value	t-value (control relative to AD)	Log2 fold change
SLC family				
SLC25A37	Solute carrier family 25 member 37: mitochondrial iron transporter	0.000128	− 5.12740967	− 0.64122381
SLC25A28	Solute carrier family 25 member 28; mitochondrial iron transporter	0.0000927	− 5.29698098	− 0.64098333
SLCO4A1	Solute carrier organic anion transporter family member 4A1; organic anion transporter	0.000103	− 5.24167279	− 1.85499048
SLC12A7	Solute carrier family 12 member 7; KCC4 K–Cl cotransporter	0.000741	− 4.23121588	− 0.67435
SLC12A6	Solute carrier family 12 member 6; KCC3 K–Cl cotransporter	0.0262	− 2.46864419	− 0.52261429
Interleukin-1-receptor family				
IL1R1	Interleukin-1 receptor	0.0185	− 2.64459755	− 0.62269524
IL1RL1	Interleukin 1 receptor-like 1	0.0000113	− 6.45879688	− 3.43624762
Carbonic anhydrase family				
CA13	Carbonic anhydrase 13	0.0152	− 2.74368279	− 0.51382619
Na–K ATPase family				
ATP1A1	Na–K ATPase transporting subunit alpha 1	0.0198	− 2.61012511	− 0.45077857
Amyloid proteins				
ABPA3	Amyloid beta precursor protein (APP) binding family A member 3	0.00154	− 3.86960931	− 0.51001667
APBB1IP	APP binding family B member 1 interacting protein	0.0116	− 2.87838249	− 0.53602143

Negative t values are for comparisons displaying gene changes that are increased in AD relative to control (i.e. decreased in control relative to AD). The same is true for negative log2FC values

The Na–K-ATPase significantly impacts CSF production [13]. Na–K-ATPase is a major pathway for Na^+^ secretion into CSF (and K^+^ uptake from CSF) [26]. The Na–K-ATPase transporting subunit α2, ATP1A2 (p = 0.04, log2FC = 0.51), and the Na–K-ATPase transporting subunit β1, ATP1B1 (p = 0.04, log2FC = 0.29), were downregulated in AD. Of all ATPase genes investigated, only ATP1A1 (p = 0.02, log2FC = − 0.45), the transporting subunit α1, was upregulated in AD.

Another gene family integral to CSF production and homeostasis is carbonic anhydrase (CA). CA catalyzes production of HCO_3_^−^ and H^+^ from H_2_O and CO_2_ [26]. HCO_3_^−^ is essential to CPE transport processes; thus proper generation of HCO_3_^−^ by CA in CPE is vital to the ability of the CP to secrete CSF [27]. Isoforms showed diverse up- and downregulation in AD. CA2 (p = 0.022, log2FC = 0.30), CA3 (p = 0.039, log2FC = 0.59), and CA4 (p = 0.0001, log2FC = 0.63) were downregulated. However, CA13 (p = 0.015, log2FC = − 0.51) was upregulated. Other CA gene transcripts were not significantly altered.

Barrier-stabilizing genes of the claudin family, CLDN5 (p = 0.04, log2FC = 0.71), CLDN18 (p = 0.011, log2FC = 0.47) and CLDN11 (p = 0.019, log2FC = 0.65), were downregulated in AD. For several other claudin genes known or purported to be involved in epithelial tight junctions, a tendency towards downregulation was observed, although results lacked significance. Also downregulated was Aβ precursor protein, APP (p = 0.014, log2FC = 0.41). However, other CP APP genes (e.g., APBA3, p = 0.0015, log2FC = − 0.51) were upregulated. Subunits and assembly factors of the mitochondrial F0F1 ATP synthase, an important source of ATP synthesis, such as ATP5L (p = 0.0004, log2FC = 0.44) and ATP23 (p = 0.0008, log2FC = 0.63) were downregulated in AD CP. We observed no significant changes in expression of aquaporin 4, and aquaporin 1 (a known passageway for passive flow of H_2_O from CPE to ventricular lumen).

Tables 3 and 4 list genes that did not display significant changes in expression levels between AD and control groups, but displayed tendencies towards upregulation and downregulation that may be of interest for future studies.

**Table 3:** Unchanged genes with possible tendency of downregulation in AD CP

Gene symbol	Gene name	p-value	t-value (control relative to AD)	Log2 fold change
SLC family				
SLC12A4	Solute carrier family 12 member 4: KCC1 K–Cl cotransporter	0.0947	1.78492648	0.23569524
SLC4A2	Solute carrier family 4 member 2: Cl–HCO3 exchanger	0.130	1.60190388	0.21841905
SLC38A3	Solute carrier family 38 member 3; system N amino acid transporter	0.223	1.27232445	0.2807
Na–K ATPase family				
ATP1B2	Na–K ATPase transporting subunit beta 2	0.0533	2.09928405	0.39634524
ATP1A4	Na–K ATPase transporting subunit alpha 4	0.684	0.41463513	0.06897143
Carbonic anhydrase family				
CA8	Carbonic anhydrase 8	0.138	1.56854024	0.50962619
CA14	Carbonic anhydrase 14	0.172	1.4334038	0.29066667
Tight junction claudins and occludin				
CLDN1	Claudin-1	0.188	1.37904737	0.35589048
CLDN2	Claudin-2	0.161	1.47424712	0.43342857
CLDN3	Claudin-3	0.0942	1.78793258	0.41007381
CLDN19	Claudin-19	0.375	0.91449198	0.32223095
OCLN	Occludin	0.799	0.25905032	0.03019762
Others				
AQP4	Aquaporin 4	0.621	0.50457389	0.18047619

Positive t values for comparisons represent gene changes decreased in AD relative to control (i.e. increased in control relative to AD). The same is true for positive log2FC values

**Table 4:** Unchanged genes with possible tendency of upregulation in AD CP

Gene symbol	Gene name	p-value	t-value (control relative to AD)	Log2 fold change
SLC family				
SLC39A4	Solute carrier family 39 member 4; ZIP4 metal ion transporter	0.806	− 0.25019879	− 0.03455714
Carbonic anhydrase family				
CA7	Carbonic anhydrase 7	0.310	− 1.05004961	− 0.16493333
CA5B	Carbonic anhydrase 5B	0.149	− 1.5231615	− 0.23130952
CA11	Carbonic anhydrase 11	0.445	− 0.78401027	− 0.1202119
Other genes				
PSEN1	Presenilin 1	0.0687	− 1.96233878	− 0.23186667
PSEN2	Presenilin 2	0.182	− 1.39857889,	− 0.1595881
AQP1	Aquaporin 1	0.367	− 0.93107284	− 0.11615238

Negative t values are for comparisons displaying gene changes that are increased in AD relative to control (i.e. decreased in control relative to AD). The same is true for negative log2FC values

The large number of significantly altered CP genes, for barrier stability, solute and H_2_O transport, and production of ATP to energize transport, makes it unlikely that CSF production is maintained normally in AD.

## Discussion

The basic requirements for CSF production are (i) transport of ions and H_2_O from CPE to the ventricular CSF space, (ii) production of energy to facilitate that active transport and (iii) an intact CP. Analysis of our Brown-Merck GEO database revealed significantly-altered gene expression in AD CP that adversely impacts the physiology necessary for CSF secretion. Genes for ion transport, HCO_3_^−^ production and barrier stability were downregulated in AD. Genes involved in inflammation and Aβ accumulation were also upregulated in AD CP. Altered mitochondrial enzyme and transporter expression, associated with decreased ATP production, reflects AD-associated metabolic- and oxidation-related defects at the BCSFB. All of these changes likely contribute to decreased CSF production in AD, though the reader must be aware that protein expression and mRNA expression are not always directly related. In addition, the reader should be aware that while fold changes are a good indicator of the magnitude of change in gene expression, they do not necessarily translate to functional significance. Specific gene expression values for each gene in control and AD cases would be useful for better understanding functional relevance of the gene expression changes detailed here; hence the provided Additional file 1 of all gene expression values.

Various lines of evidence suggest that Aβ accumulation in AD is a problem of clearance rather than overproduction [30, 31]. CSF production and turnover are part of the clearance mechanisms of the CNS. Communication with the extracellular fluid (ECF) space and the paravascular space [32, 33] allows the CSF pathway to function efficiently as a clearance pathway.

Upregulated Aβ precursor protein binding family protein APBA3 in CP is consistent with plaque buildup [33, 34]. Aβ retention in AD CP [35] and ECF favors oligomerization and deposition, weakening BCSFB tight junctions while increasing levels of inflammatory cytokines and matrix metalloproteinase [21]. Aβ burden in the brain and its barriers may trigger microglia activation due to brain injury and promote reactive astrocytes. The transformation of astrocytes into neurotoxic reactive cells, through increased secretion of interleukin-1α, tumor necrosis factor α, and complement C1q leads to self-perpetuating, widespread neuronal death in AD [36].

Unchecked expression of inflammatory mediators in response to brain tissue damage and barrier disruption [37] increases activation of brain microglia, and promotes invasion of additional immune cells through BCSFB into CSF and brain. CP upregulation of interleukin-1 receptor (IL1R) and interleukin-1 receptor like 1 (IL1RL1) in AD coincides with increased IL-1 secretion by activated microglia. Among the cytokine families, activated IL1R promotes acute and chronic inflammation [38]. Antagonists of IL1R have potent anti-inflammatory effects [39].

Along with Aβ binding protein and IL1R upregulation, downregulated claudin-5, claudin-11 and claudin-18 may contribute to barrier degradation in AD. Claudin-5 is an important structural component of tight junction strands [40], and a gatekeeper protein regulating paracellular transport at BCSFB tight junctions [20]. Information is lacking for CP claudin-18, although high claudin-18 expression occurs in lung alveolar epithelial tight junctions [41]. Claudin-11 is an essential component of myelin, and claudin-11 null mice lack myelin sheath tight junctions [42]. Furthermore claudin-11 downregulation increases BCSFB permeability to FITC-dextran [43]. Other claudin mRNA examined did not display significant changes in AD CP.

Increased BCSFB transcellular and paracellular permeability in AD disrupts the CP–CSF secretory, synthetic, and transportation functions [3]. CSF carries essential nutrients and ions, at homeostatic concentrations, into brain [44]. This enables CSF control of temperature, blood pressure, and pH [6].

In the context of ion transport and CSF formation, it is pertinent to first evaluate CP mitochondria in AD. AD hippocampal and CP cells are deficient in the mitochondrial enzyme cytochrome c oxidase, complex IV of the electron transport chain [45]. Mitochondrial dysfunction also links to autophagy in AD. This inability to degrade defective macromolecules and organelles links with harmful neuronal lipofuscin buildup [46]. Excess lipofuscin in aging and in the AD CP is problematic for Aβ plaque formation [47, 48].

The key enzyme for ATP synthesis is mitochondrial ATP synthase. Choroidal ATP synthase damage indicates depressed ATP synthesis in AD. Even if ion transporters are intact, active transporters function inefficiently with diminished ATP (e.g., Na–K ATPase, an important route for Na efflux from CPE [14], requires ATP hydrolysis for Na–K exchange). Thus an important consequence of alterations in ATP synthase subunit and assembly factor expression may be disrupted ATP-dependent active transport of solutes across CPE–CSF in AD, leading to impaired solute concentration gradients that are integral for CSF production. Turning to specific ion transporters, Na–K-ATPase is a heterodimer of an α and β subunit. Four α subunits and three β subunits exist in mammals [49]. Na–K ATPase is at the apical CPE and CSF secretion is reduced by inhibiting Na–K-ATPase [26]. This is predictable given the Na–K-ATPase role in primary active Na^+^ secretion into CSF (and K^+^ removal from CSF). ATP1A1, found to be the dominant catalytic subunit of the Na–K ATPase in mouse studies [50], was mildly upregulated in AD CP while other lesser-expressed subunits ATP1A2 and ATP1B1 were downregulated. Upregulation of ATP1A1 in AD CP, if it is the same in humans, by itself would suggest increased Na–K pumping and increased CSF production. However, given decreased CSF production in AD [1], the contribution of the Na–K ATPase may not be as relevant as other solute transporters in CSF production disturbances in AD.

The Na–K–Cl cotransporter NKCC1, encoded by the SLC12A2, has an important role in solute transport. NKCC1 is at the apical CPE [51]. Steffensen and colleagues [52] suggest that NKCC1 is responsible for ~ 50% of CSF production. This finding attributes a central role for NKCC1 in enabling H_2_O to flow from CPE to CSF. Earlier theories proposed a simple osmotic model of NKCC1 coupling to an aquaporin [53, 54]. We observed no significant change in AQP1 and AQP4 in AD CP. However, CSF production declines by ~ 20% in AQP1 knockout (KO) mice [16], different from the ~ 50% suggested by Steffensen et al. who theorized that H_2_O accompanies ion flux directly through NKCC1 [52]. Considering the likely role of NKCC1 to facilitate H_2_O flow for CSF production, decreased NKCC1 mRNA in AD versus control CP agrees with generally-decreased CSF production and impaired CSF dynamics measured in AD [1]. We conclude that CSF dynamics diminution in AD is not mainly attributable to reduced aquaporin expression. Therefore, by deduction, altered H_2_O movement across CPE in AD is more likely related to lower NKCC1 mRNA (Table 1). Still, further analysis of NKCC1 transcript vs. protein expression in CP is needed to reconcile disparate findings [55] for AD.

Bicarbonate transport across CPE sustains CSF production [56]. Many transporters utilize HCO_3_ gradients to move cations and anions into and out of CPE. Acetazolamide inhibition of CA distorts pH gradients among CPE, CSF and brain [29]. In addition, Vogh and colleagues established that CA inhibitors reduce CSF production by > 50% [57]. This suggests a sizeable portion of CSF production depends on cell-produced HCO_3_.

Na-dependent Cl–HCO_3_ exchange by CP SLC4A10 gene is at the basolateral face. SLC4A10 mediates influx of a Na^+^ and HCO_3_ ion per efflux (into CP interstitium) of one Cl^−^ ion [26]. SLC4A10 KO mice showed decreased ventricular volume, likely from reduced CSF production [58]. There was also microvilli attenuation and CPE cell enlargement in SLC4A10 KO mice. Given this study, loss of SLC4A10 in AD would be expected to depress CSF production. The Na–HCO_3_ cotransporter SLC4A5, downregulated in AD, is in apical CPE. There it mediates transport of three HCO_3_ and one Na^+^ from CPE into CSF. This stoichiometry/vector supports a Na–HCO_3_ role to counter CSF acidity while promoting CSF formation [59]. SLC4A5 KO mice results resembled SLC4A10 KO mice in one study: decreased lateral ventricular volume, reduced intracranial pressure, and altered CPE structure [60]. However, a second SLC4A5 study, utilizing a different KO mouse, caused arterial hypertension but no altered ventricular volume [61]. Further research needs to clarify the CSF-supporting role of SLC4A5, although like SLC4A10, decreased expression intimates reduced CSF production in AD.

Essential to HCO_3_ transport is carbonic anhydrase activity. CA2, CA3, and CA4 were downregulated in AD. CA13, though, was upregulated. The CA’s generate HCO_3_ and H^+^ ions from H_2_O and CO_2_ [26, 29]. CA2, CA3 and CA13 are cytosolic while CA4 is tethered by a membrane anchor [62]. Of particular significance with respect to AD and CSF dynamics is the CA isoform CA2, due to its high catalytic activity and efficacy in proton shuttling [63]. High rates of HCO_3_ production by CA2 allow regulation of blood pH to preserve homeostasis. In kidneys, CA2 and CA4 associate with HCO_3_-anion transporters and proton antiporters, directly coupling HCO_3_-synthesis to ion exchange [62]. If a parallel system exists in CP, any downregulated CA2 and CA4 in AD CP would directly disrupt the action of HCO_3_-anion transporters, decreasing active solute transport and depressing CSF formation.

A limitation of this study is the focus on pathways we considered important for CSF production and the structural integrity of the CP. We therefore did not examine *every* gene in the Brown-Merck GEO database that possibly impacts CSF production. Unknown genes omitted by us may be found by others in future CSF dynamics analyses to be important. In an earlier study, Bergen et al. analyzed gene expression profiles of control and AD subjects based on RNA extracted from laser-dissected CPE cells [20]. Mining of their database may yield additional new insights. Comparison of their data with ours was not possible due to methodological differences. However, Stopa et al. analyzed common gene transcripts controlling CSF dynamics/homeostasis in both databases: Netherlands versus Brown-Merck. They reported ~ 70% agreement, isolated CPE versus CP tissue, with Bergen et al. [20, 24].

## Conclusions

We identified multiple genes involved in CSF production that differed in expression between AD and control CP. Many ion transporters that impact solute and H_2_O transport and fluid dynamics were downregulated in AD. Importantly for many choroid epithelial processes, F0F1 ATP synthase was downregulated; this fits a diminished energy supply for choroidal transport. Genes that maintain CP membrane tight junctions had decreased expression. Upregulated CP genes in AD included those mediating chronic inflammation and neurodegeneration. Each altered gene transcript in this study is a potential candidate to explain the altered CSF production observed clinically in AD. Demonstration of causal relationships may lead to new therapeutic targets for AD aimed at bolstering CSF production and turnover.

## Additional file

**Additional file 1.** Gene expression values.

## Abbreviations

Aβ: amyloid beta
AD: Alzheimer’s disease
APP: amyloid precursor protein
AQP1: aquaporin 1
ATP: adenosine triphosphate
BBB: blood–brain barrier
BCSFB: blood–CSF barrier
CA: carbonic anhydrase
CLDN: claudin
Cq1: complement factor q1
CSF: cerebrospinal fluid
CP: choroid plexus
CPE: choroid plexus epithelium
GEO: Gene Expression Omnibus
IL-1: interleukin-1
KCC: potassium–chloride cotransporter
KO: knockout
NKCC: sodium–potassium–chloride cotransporter
RNA: ribonucleic acid
SLC: solute carrier
TNFα: tumor necrosis factor alpha
TTR: transthyretin
